# Social Determinants of Health Associated with the Use of Screenings for Hypertension, Hypercholesterolemia, and Hyperglycemia among American Adults

**DOI:** 10.3390/medsci9010019

**Published:** 2021-03-23

**Authors:** Tran Nguyen, Amanda Barefield, Gia-Thien Nguyen

**Affiliations:** 1 College of Allied Health Sciences, Augusta University, Augusta, GA 30912, USA; ABAREFIELD@augusta.edu; 2 Medical College of Georgia, Augusta University, Augusta, GA 30912, USA; GNGUYEN@augusta.edu

**Keywords:** chronic disease prevention, hypertension screening, hypercholesterolemia screening, hyperglycemia screening, secondary preventive services

## Abstract

National and international health guidelines have recommended measurements of blood pressure, blood cholesterol, and blood glucose as the first step in detecting hypertension, hypercholesterolemia, and hyperglycemia, respectively. These chronic conditions are modifiable risk factors for chronic diseases such as obesity, diabetes, and cardiovascular disease. Social determinants of health (SDoHs) have contributed to persistent chronic condition disparities in the United States. This study identified SDoHs associated with the use of screening services for hypertension, hypercholesterolemia, and hyperglycemia by analyzing data from the 2019 United States National Health Interview Survey. Examined SDoHs consisted of demographic characteristics, socioeconomic status, and health care utilization. Age, gender, education, annual income, health coverage, and usual care source were positively associated with the odds of receiving secondary preventive services. There was a marginal significance among race/ethnicity and employment status in association with the odds of receiving secondary preventive services. This study’s findings inform health educators and providers, public health professionals, and policymakers to fund, plan, and coordinate services and interventions accordingly to improve the population’s quality of life and lengthen lifespan by promptly diagnosing and treating these diseases.

## 1. Introduction

Hypertension, hypercholesterolemia, and hyperglycemia are critical modifiable health risk factors of chronic diseases, such as obesity, diabetes, and cardiovascular disease [1]. In the United States (U.S.), chronic diseases are not only the leading cause of death and disability, but also the driver of the country’s $3.5 trillion in annual healthcare expenditures [2]. Moreover, individuals with one or more chronic diseases appear to be at higher risk for severe outcomes in infectious diseases if they ever contract one [3]. A recent study from the U.S. Centers for Disease Control and Prevention (CDC) estimated more than half (51.8%) of American adults had at least one chronic disease, and 27.2% had more than one [4]. Fortunately, many chronic diseases are preventable. The concept of the secondary prevention of screening for risk factors among asymptomatic individuals, to halt the development of the disease, has been of interest as one of the essential tools of modern public health and preventive medicine [5]. Screening tests that detect chronic conditions at an early stage can increase the prevention and management of the diseases, improve health outcomes, decrease treatment costs, and reduce chronic diseases’ economic impact [6,7]. As the first step in detecting these conditions, national and international health guidelines have recommended blood pressure measurements every year for hypertension, blood cholesterol every five years for hypercholesterolemia, and blood glucose every three years for hyperglycemia [8,9,10]. These blood tests are simple, non-invasive, cost-effective, and can be performed at the primary care centers’ convenience.

The term “social determinants of health” (SDoHs) refers to conditions that people are born, grow, live, learn, and work with, affecting their well-being [11]. Public health literature reveals that SDoHs have contributed to the disparities of chronic diseases and, their secondary prevention [12,13,14,15]. In an effort to reduce health disparities, the Patient Protection and Affordable Care Act (ACA) was passed into law in 2010 to provide healthcare access to all Americans. Due to the importance of preventive care, some provisions expand access to adequate health insurance coverage to include preventive services to reduce chronic diseases’ risk factors [16]. Nevertheless, health literature has demonstrated that secondary preventive services are underutilized, despite their evidence-based benefits and no cost-sharing responsibilities under ACA [17].

Many existing health literature studies have analyzed preventive service disparities pre- and post- ACA era; however, most of them focused on the screening of different cancer types [18]. This study aimed to examine utilization of the recommended blood tests as screenings for hypertension, hypercholesterolemia, and hyperglycemia among American adults, to prevent obesity, diabetes, and cardiovascular diseases. The analyses further identified SDoHs associated with the use of these preventive services. We sought to answer the research questions:

1. To what extent do American adults undergo the recommended screenings for hypertension, hypercholesterolemia, and hyperglycemia?
2. Would the associations of SDoHs on screening status be congruent with the literature in the pre-ACA era?
3. Can SDoHs predict the screening status of these conditions?

The contribution of this research is three-fold. Firstly, this study adds to the growing knowledge of health literature about factors associated with the use of recommended preventive services in the post-ACA era. Secondly, the findings potentially guide public health interventions targeting individual and community levels to improve the prevention of chronic diseases to close the gap in health and life quality among Americans. Thirdly, we provide useful information to policymakers for decision making to bolster programs that will benefit the public’s well-being.

## 2. Methods

### 2.1. Data Source

This research utilized the design of a quantitative method that employed a cross-sectional observation study based on secondary data, the 2019 National Health Interview Survey (NHIS). The NHIS, conducted annually by the National Center for Health Statistics (NCHS), is a nationally representative survey of non-institutionalized civilians in the U.S. and covers a broad range of health topics. The NHIS uses a multistage sampling method with a new sample of respondents interviewed each year. There are two main components in the core questionnaire, the Sample Adult and the Sample Child. Families are identified in each household. One “sample adult” aged 18 years or more, and one “sample child” aged 17 years or less are randomly selected from each household. The Research Ethics Review Board of the NCHS and the U.S. Office of Management and Budget approved the NHIS. All respondents provided oral consent before participation [19].

This study analyzed the Sample Adult component to examine SDoHs associated with recommended screenings for hypertension, hypercholesterolemia, and hyperglycemia. In the 2019 NHIS public-use data files, 31,997 out of 35,365 adults completed the survey, achieving a conditional-response rate of 90.5%. Since this study focused on preventing chronic conditions, those who had been told of having hypertension, hypercholesterolemia, and hyperglycemia were excluded from the study sample. The final sample consisted of 16,680 subjects.

### 2.2. Measures

This study had three dependent variables, operationalized by the survey items, where respondents were asked when was the last time they had measurements of blood pressure, blood cholesterol, and blood glucose checked by a doctor, nurse, or other health professional. Outcome choices were: “never”, “within the past year”, “within the last 2 years”, “within the last 3 years”, “within the last 5 years”, “within the last 10 years”, “10 years ago or more”. Based on a recommendation from the national and international guidelines, dependent variables were recoded to dichotomous, as blood pressure checked within the last year (no or yes), blood cholesterol checked within the last 5 years (no or yes), and blood glucose checked within the last 3 years (no or yes). Hereafter, the term “blood tests” indicates the tests performed within the recommended timelines.

Social determinants of health (SDoHs) served as independent variables that constituted demographic characteristics, economic status, and health care utilization. Demographic characteristics comprised: age (18–39 years, 40–60 years, or 61 years and above), sex/gender (male or female), race/ethnicity (Hispanic, non-Hispanic White, non-Hispanic Black, non-Hispanic Asian, non-Hispanic American Indian and Alaska Native, or other non-Hispanic groups and multiple races), and marital status (single/never married or married/living with a partner). The predictor measures of economic status consisted of education (less than high school, high school/equivalent, or some college/college degree), employment status (non-employed or employed), and annual household income (below $35,000, 35,000–75,000, or above $75,000). Health care utilization included health care coverage (no or yes) and having a usual source of routine or preventive care (no or yes). All variables were self-reported. To test whether our data presented the issue of multicollinearity, we computed the binary Pearson correlation coefficients for all independent variables. Our data exhibited low collinearity between independent variables with the correlation coefficients of less than |0.3|. Table 1 presents the output of our correlation study.

### 2.3. Data Analysis

IBM SPSS^®^ Statistics version 25.0 (IBM Corporation Armonk, New York, NY, USA) was the data analysis statistical software used for this study. Descriptive statistics of variables were computed as appropriate. For each dependent variable, multivariate logistic regression was performed to determine the relationship between SDoHs and the recommended blood test. The adjusted odds ratios (AOR) and p-values were evaluated to assess the percentage estimates’ variability and make comprehensive comparisons within and across groups. All statistical tests were at the significance level of α = 0.05. This study utilized the sampling weights developed by the NCHS in all of the analyses to reflect the multistage sampling design of the survey. Additional information about the survey methods is available on the NCHS website [19].

## 3. Results

Table 2 shows the descriptive demographic characteristics, economic status, and health care utilization of the study sample. According to respondents’ self-report, approximately 84% checked blood pressure within the last year, 87% checked blood cholesterol within the last five years, and 79% checked blood glucose within the last three years.

Table 3 shows the associations between examined SDoHs and the likelihood of respondents receiving the blood tests. The associations generally agreed across the three tests studied. Among age groups, the older groups were more likely to check their blood tests compared to the younger ones, except for the 40–60-year-old age group, who were less likely to check their blood pressure, compared to the 18–39-year-old age group. In contrast to their male counterparts, females were more likely to check their blood tests. There was a marginal significance among race/ethnicity in association with the odds of checking their blood tests. Compared with Hispanic, non-Hispanic Whites were less likely to check their blood cholesterol and glucose, while non-Hispanic Blacks were more likely to check their blood pressure and glucose. Non-Hispanic Asians and other groups/multiple races were almost equally, less likely to check blood cholesterol and glucose. Non-Hispanic AIAN showed no significant association with checking their blood tests. When compared with the single, the married, or living with a partner was were more likely to check their blood tests.

With regard to education level, the some college or college degree group, exhibited significant associations with the odds of checking all three blood tests. The high school graduate or equivalent group, showed significant association in checking their blood cholesterol, but not blood pressure or glucose levels. The employment status showed a significant association with checking blood glucose, but not blood pressure or cholesterol. Employed individuals were less likely to check their blood glucose in comparison to unemployed individuals. The annual household income had significant positive associations with the odds of checking the blood tests. Higher incomes demonstrated higher odds. In contrast to individuals with no health care coverage, those who had health care coverage were more likely to check their blood tests. Participants who reported having a usual source of care were also more likely to check their blood tests subsequently, than those without one.

## 4. Discussion

Hypertension, hypercholesterolemia, and hyperglycemia are risk factors shared among obesity, diabetes, and cardiovascular disease. This study attempted to determine the prevalence of screenings for these conditions, identify SDoHs associated with adopting these preventive services in the U.S, and arbitrate whether the associations of SDoHs on screening status are congruent with the literature on health screenings in the pre-ACA era. Our results show high rates for the utilization of the blood tests, ranging from 78.7% for blood glucose to 86.6% for cholesterol. The high rates may be credited to the ACA’s implementation in 2010. The law links recommendations from the U.S. Preventive Service Task Force (USPSTF) and requires insurance providers to cover the recommended services without copayments, deductibles, or co-insurance. That action made obtaining screenings easier and free of financial obligation for Americans.

The findings of SDoHs associated with the aforementioned conditions’ screening status are mostly congruent with the literature on health screenings in the pre-ACA era. Age has significantly been associated with the odds of utilizing the blood tests. This finding might be attributable to the increased risk of acquiring chronic diseases with increased age [20]. Gender is a significant predictor of screening status for hypertension, hypercholesterolemia, and hyperglycemia. Females are more likely to receive the recommended blood tests. The results are consistent with existing studies, demonstrating that females are more likely to participate in general health screenings than their male counterparts [21]. Individuals who were married or living with a partner are more likely than those who were, never married or single to check their blood tests. Literature has linked marriage to better health outcomes, even though mechanisms for this observance were unclear. Scholars suggested that marital partners might be motivated to maintain their health because of the obligations to other family members who depend on them for economic security and social support [22].

Our results show a marginal significance among race/ethnicity in association with the likelihood of screening for hypertension, hypercholesterolemia, and hyperglycemia. Empirical studies focusing on the impact of race/ethnicity on health disparities indicated that race/ethnicity was a significant predictor for adults’ preventive behavior [22,23,24]. In this study, non-Hispanic Blacks are more likely than other groups to check their blood pressure and glucose. The finding differs from what had been reported in the literature as non-Hispanic Blacks are less likely to seek routine or preventative care than their racial/ethnic counterparts due to limited engagement with the health system [25]. Another surprising result is that non-Hispanic Whites are less likely than Hispanics to check their blood cholesterol and glucose. This finding contradicts the common knowledge that non-Hispanic Whites have a higher prevalence in health services utilization than other races. Perhaps, the increase in community health promotion programs during the past decade has fostered patient empowerment that ensures patients from minority groups understand and demand preventive health screenings to eliminate health disparities [26]. This area warrants further research, better focused on investigating a notable delineation of the effect of these factors.

Education level demonstrates significant association with receiving the blood tests. Adults with some college or college degree are possibly well informed and more knowledgeable of preventive health services’ benefits. Chronic disease prevention requires individuals to adopt a proactive stance on gathering information. In addition, whereas underprivileged individuals tend to seek help only when a need emerges [27]. Additionally, disadvantaged individuals may not be well informed about the ACA benefits. Household income has significant positive associations with receiving the blood tests; higher incomes lead to higher odds. There is a plausible explanation that low-income individuals tend to seek help for only urgent matters, as they may be unable to afford the cost of missing a day’s pay or, lack transportation [28].

The finding that employed individuals are less likely to check their blood tests than unemployed ones is quite surprising. Previous studies that examined the preventive behavior in breast and cervical cancers found that employed individuals had a significant favorable influence on the likelihood of receiving the screenings [29]. Employment and healthcare access are positively correlated, since employed individuals often have health insurance through their employers [30,31,32]. Two reasons may explain this phenomenon: increasing workplace wellness programs, and the implementation of the ACA. Recognizing the importance of employee well-being and other economic benefits, many employers provide wellness programs to their employees, including several preventive services [33]. Under the ACA, Americans without employment can obtain subsidized healthcare insurance through the federal government’s health care exchange.

Having a usual source of care is the most critical determinant of the screenings for hypertension, hypercholesterolemia, and hyperglycemia, followed by healthcare coverage. Individuals who have a usual source of care are more likely to receive recommended preventive services. In contrast, having healthcare coverage provides the mean for one to utilize primary and preventive care services. These two factors are well documented in health literature as strong determinants of better health outcomes [34,35]. Nevertheless, the rate of having a usual source of care (85.9%) that is lower than the rate of having healthcare coverage (87.6%) poses an important issue. Americans underutilize their healthcare benefits, which limits their ability to achieve the highest level of health. Future research may look further into impeding and facilitating factors for the best use of healthcare coverage. In addition, frequent blood tests benefit early diagnosis and the managing of chronic diseases for better health outcomes and reduced treatment costs. Health insurance providers may proactively establish policy bolstering the use of primary care sources.

While we reported some informative findings, our study had several strengths and limitations. One strength was the use of NHIS data that represented a cross-section of the entire U.S. population. Moreover, NHIS data provides detailed and up-to-date measures of health status, and the adoption of preventive services. This recent and national representative sample enhances our study’s generalizability. The limitations include the reliance on self-reported data, which may lead to under- or over-reporting preventive actions and a subject-to-recall bias. Further, by using a cross-sectional study design, this research cannot establish the temporal relationship between SDoHs and the odds of receiving the screenings; thus, there is no causation.

## 5. Conclusions

Access to preventive services can avert both diseases and premature deaths. Even though the prevalence of these American services has increased under the ACA, there remain disparities by SDoHs. The new approach to public health emphasizes section collaboration, environmental policy, and systems-level actions that directly affect healthcare social determinants. Public health practitioners and educators may customize health promotion programs, depending on the population served, to educate about the benefits of preventive services and the ACA. Health insurance policymakers need to look for new approaches to bolster the use of primary care benefits. Future research is beneficial to investigate the effects of race, ethnicity and workplace wellness programs on adopting preventive services, and the utilization of impeding, facilitating healthcare factors to improve their effectiveness.

As a final thought, the burden of chronic diseases is currently at the forefront of our nation’s attention. During this research, the COVID-19 pandemic continued to rampage globally, resulting resulted in the loss of over 500,000 American lives. The U.S. CDC reported that only 6% of American COVID-19 deaths between 12 February and 28 March 2020, were attributable to the infection alone, while 94% were associated with other chronic conditions or comorbidities [3]. The most frequently reported diseases were diabetes, chronic lung disease, and cardiovascular disease. Prevention and management of chronic diseases are approaches to reducing health disparities, that can help achieve health equity, and prepare the nation to face future epidemics. COVID-19 is not likely to be the last one.

## Figures and Tables

**Table 1:** Correlation output of independent variables (N = 31,997): NHIS 2019.

	Age	Sex	Race	Marital Status	Education	Employment	Annual House-hold Income	Health Coverage	Usual Source of Care
**Age**	1	0.039 **	−0.024 **	0.052 **	−0.009	−0.321 **	−0.032 **	0.127 **	0.127 **
**Sex**	0.039 **	1	0.010	0.014	0.068 **	−0.122 **	−0.033 **	0.060 **	0.148 **
**Race**	−0.024 **	0.010	1	−0.039 **	0.106 **	−0.032 **	−0.019 *	0.070 **	0.026 **
**Marital Status**	0.052 **	0.014	−0.039 **	1	0.065 **	0.074 **	0.269 **	0.036 **	0.056 **
**Education**	−0.009	0.068 **	0.106 **	0.065 **	1	0.123 **	0.230 **	0.223 **	0.095 **
**Employment**	−0.321 **	−0.122 **	−0.008	0.074 **	0.123 **	1	0.191 **	−0.023 **	−0.058 **
**Annual Household Income**	−0.032 **	−0.033 **	−0.019 *	0.269 **	0.230 **	0.191 **	1	0.150 **	0.080 **
**Health Coverage**	0.127 **	0.060 **	0.070 **	0.036 **	0.223 **	−0.023 **	0.150 **	1	0.274 **
**Usual Source of Care**	0.127 **	0.148 **	0.026 **	0.056 **	0.095 **	−0.058 **	0.080 **	0.274 **	1

NHIS = National Health Interview Survey. ** Correlation is significant at the 0.01 level (2-tailed). * Correlation is significant at the 0.05 level (2-tailed).

**Table 2:** Descriptive of the study sample (N = 31,997): NHIS 2019.

**Dependent Variables**		**n ^1^** **(Not Weighted)**	**Percentage** **(Weighted)**
Hypertension screened within the past year (Blood Pressure)	No	2694	16.3
	Yes	13,791	83.7
Hypercholesterolemia screened within the past 5 years (Blood Cholesterol)	No	2115	13.4
	Yes	13,701	86.6
Diabetic screened within the past 3 years (Blood Glucose)	No	3367	21.3
	Yes	12,422	78.7
**Independent Variables**		**n ^1^** **(Not Weighted)**	**Percentage** **(Weighted)**
Age	18–39 years	8147	48.8
	40–60 years	5682	33.5
	61+ years	2951	17.7
Sex	Male	7485	44.9
	Female	9192	55.1
Race/Ethnicity	Hispanic	2567	15.4
	Non-Hispanic White	10,990	65.9
	Non-Hispanic Black	1636	9.8
	Non-Hispanic Asian	1051	6.3
	Non-Hispanic AIAN	106	0.6
	NH other groups or multiple races	330	2.0
Marital Status	Single	7252	44.6
	Married or living with a partner	9014	55.4
Education	Less than high school	1217	7.3
	High school graduate or equivalent	4035	24.3
	Some college or college degree	11,334	68.4
Employment	Unemployed	4243	26.1
	Employed	12,029	73.9
Annual household income	<$35,000	4099	35.5
	$35,000–$75,000	5208	45.1
	>$75,000	2236	19.4
Health Coverage	No	2064	12.4
	Yes	14,559	87.6
Usual Source of Care	No	2334	14.1
	Yes	14,255	85.9

NHIS = National Health Interview Survey. SD = Standard Deviation. AIAN = American Indian and Alaska Native. ^1^ The total not added up to N indicated missing observations.

**Table 3:** Multivariate logistic regression analyses of sociodemographic characteristics associated with the utilization of hypertension, hypercholesterolemia, and diabetic screening among American adults: NHIS 2019.

Dependent Variables		B.P. Screening	BC Screening	BS Screening
		AOR	AOR	AOR
**Age**	18–39 years	--	--	--
	40–60 years	0.864 *	1.736 **	1.509 **
	61+ years	1.212 *	2.807 **	2.463 **
Sex	Male	--	--	--
	Female	2.039 **	1.595 **	1.653 **
Race/Ethnicity	Hispanic	--	--	--
	Non-Hispanic White	1.104	0.520 **	0.545 **
	Non-Hispanic Black	1.423 **	1.205	1.267 *
	Non-Hispanic Asian	0.860	0.511 **	0.585 **
	Non-Hispanic AIAN	1.029	0.698	0.650
	NH other groups or multiple races	1.106	0.609 *	0.509 **
Marital Status	Single	--	--	--
	Married or living with a partner	1.159 *	1.141 *	1.245 **
Education	Less than high school	--	--	--
	High school graduate or equivalent	1.083	1.232 *	1.157
	Some college or college degree	1.395 **	1.874 **	1.385 **
Employment	Unemployed	--	--	--
	Employed	0.914	0.882	0.868 *
Annual household income	<$35,000	--	--	--
	$35,000–$75,000	1.202 *	1.316 **	1.291 **
	>$75,000	1.146	1.547 **	1.376 **
Health coverage	No	--	--	--
	Yes	2.255 **	1.963 **	1.824 **
Usual Source of Care	No	--	--	--
	Yes	4.095 **	3.398 **	3.571 **

NHIS = National Health Interview Survey. BP = blood pressure. BC = blood cholesterol. BS = blood glucose. AOR = Adjusted odds ratio. AIAN = American Indian and Alaska Native. * = significant at 0.05. ** = significant at 0.01.

## Data Availability

Publicly available dataset was analyzed in this study. This data can be found here: https://www.cdc.gov/nchs/nhis/2019nhis.htm (accessed on 22 March 2021).
